# Health care providers’ perspectives on the need for palliative care in Upper Egypt: a descriptive exploratory study including children and adult patients

**DOI:** 10.1186/s12904-024-01469-5

**Published:** 2024-06-15

**Authors:** Atiat Osman, Savannah Gail Horvick, Nancy Dias

**Affiliations:** 1 Lecturer of Pediatric Nursing, Pediatric Nursing Department, Faculty of Nursing, South Valley University, Qena, Egypt; 2 Savannah Gail Horvick, BSN, East Carolina University College of Nursing, 2205 W 5th St, Greenville, RN, NC USA; 3 College of Nursing/Dept. of Nursing Science, East Carolina University, 4165F Health Science Building, Greenville, NC 27858 USA

**Keywords:** Palliative care, Needs assessment, Pediatric palliative care, Cancer

## Abstract

**Background:**

Only four centers in Egypt provide Palliative Care (PC) for adult cancer patients and one provides care for pediatric cancer patients. While PC is not widely utilized in Egypt, this study aims to assess patients’ need for PC from the providers’ perspective. The primary objectives were to assess providers’ knowledge about PC, understand patients’ needs, and compare children’s and adults’ needs for PC.

**Methods:**

A descriptive exploratory design was utilized. Patients were recruited from a cancer center in Qena Governorate, Egypt. All 108 nurses and physicians in the cancer center were interviewed to assess their perspectives about PC and patients’ need for it.

**Results:**

Of the 108 care providers, more than 60% of the providers were not familiar with the concept of PC and did not participate in related activities, and more than 77% did not receive any training on the topic. All the providers reported there is no specific policy for end-of-life care. More than 60% of the providers responded that their patients do not need PC as the providers believe that PC provided only for end-stage patients. 50% of the providers see that PC has benefits such as pain relief and symptom management. No major differences were noted between pediatric and adult PC needs.

**Conclusion:**

The results of this study provide foundational evidence of providers’ lack of experience with and understanding of palliative care. This deficit is creating a barrier to providing palliative care in Egypt.

**Supplementary Information:**

The online version contains supplementary material available at 10.1186/s12904-024-01469-5.

## Introduction

Palliative care (PC), as defined by the World Health Organisation (WHO) [1], is a method that enhances the quality of life for patients and their families dealing with issues related to life-threatening illness by preventing and relieving suffering through early detection, precise assessment, and treatment of pain and other issues related to the body, mind, and spirit. Palliative care can improve the quality of life for sick patients and their families [2, 3]. The WHO reported that the quality of life of at least 100 million people would be improved if PC were accessible to all [3]. Similarly, according to the Global Atlas of Palliative Care, half a million children need PC internationally [4]. Thus, all members of the healthcare team can benefit from using a PC philosophy and approach in various avenues of care. Palliative care expertise has historically been derived from working with cancer patients, however, there has been a noticeable increase in the understanding and acceptance that PC may also benefit those with other life-limiting illnesses. PC is becoming more widely acknowledged as a basic human right and as a crucial part of comprehensive treatment for the entire life course [5] and thus needs to be integrated in the healthcare system in Egypt.

Egypt has the highest population in the Arab region, and the third-highest in Africa [6, 7], exceeding 100 million. Initiating the PC service requires awareness of the cultural, theological, and ethical background as the care should be sensitive to cultural differences [8]. It embraces physical, emotional, social, and spiritual elements of care, maintaining direct contact to recognize patient needs, while focusing on the enhancement of quality of life for a patient and their family [9–11]. Currently, Egypt has four recognized medical centers that provide palliative care services to patients [12], however, only one specializes in childhood cancer, and all three centers are located in the capital, Cairo. Approximately 40% of the Egyptian population is children, and cancer is the second leading cause of death among children [13, 14]. According to Egypt’s Pediatric Oncology Care Assessment, the survival rate for childhood cancer is only 40% [15, 16]. In contrast, the survival rate in developed countries, including the United States of America and England, is 63.4% [17]. A PC model that is suitable for the needs, culture, and resources in Egypt needs to be developed [18]. The assessment of PC is the first step in implementing such a program in Egypt, and it should aim to understand healthcare providers’ knowledge, interests, and perspectives of PC.

### Aim of the study

The study’s aim is to identify the need for a palliative care program through the perspective of healthcare providers to improve the availability and delivery of PC services including adult and pediatric patients in Egypt.

Primary objectives:

1. Assess provider’s knowledge of palliative care.
2. Explore providers’ perspectives regarding the need for palliative care programs.
3. Examine and compare the difference between adult and pediatric PC needs.

## Methods

### Design

a descriptive exploratory research design was utilized to address the study’s aim.

Measure: Data was collected using three tools that entailed two surveys and semi-structured interview:

1. Tool one (survey one): Sociodemographic Data

Includes information about the providers such as profession, specialty, and years of experience (see supplementary file).

2) Tool two: Providers’ knowledge of Palliative Care semi-structured interview

A study-specific semi-structured interview guide was developed to collect data for the 1st and the 2nd objectives. It was based on an extensive literature review, using previously developed needs assessment instruments for assessing PC as a guide [19–24]. It focused on assessing the provider’s knowledge of PC. In addition, some questions assess the providers’ perspective about patients’ need for PC (see supplementary file). Three nursing faculty experts in the oncology field reviewed the interview guide and provided feedback. Appropriate revisions were made based on their feedback.

3) Tool three (survey two): needs Assessment Tool (NAT: PD-C)

A Needs Assessment Tool: Progressive Disease-Cancer (NAT: PD-C) was used to identify specific needs for PC (2nd objective). The previous version (NAT) of this tool was used with permission from the original author [20]. It entails 19 questions with “yes” or “no” responses. These 19 questions were divided into three parts; (a) Patient well-being section contains 12 questions, (b) Ability of caregiver/family to care for the patient section contains 5 questions, and (c) caregiver well-being contains two questions. The form of NAT was used without a cut-off score.

**Setting**: The study was conducted at a specialized oncology center launched in 2016 to serve patients from three different Governorates in Egypt: Qena, Red Sea, and New Valley. The center has a capacity of 112 beds with 8 intensive care beds, serving about 40,000 adult and child patients annually, providing chemotherapy, surgical procedures, and outpatient clinics. The Pediatric Oncology Department opened in September 2019 with a capacity of approximately 10 beds and a total of 7 nurses and 6 physicians serving more than 200 children annually.

### Sample

All healthcare providers (nurses and physicians) in the Cancer center were included in the study regardless of age, specialty, and department.

### Data collection procedures

Approval from the Ethics and Research Committees from the study site was obtained to implement this study. An official letter, a copy of the proposal including used tools, and a brochure containing brief information about PC including definitions and benefits were handed over to the cancer center manager to obtain his permission to start data collection. Each provider was asked to participate at their convenience. Data collection started with questions that allowed the participants to elaborate on their understanding of PC with questions like, *Are you familiar with palliative care?* And *What does palliative care mean to you?* If providers were unable to give a correct definition for PC, they were provided with a brochure that contained additional information. Also, more questions were asked to assess their opinions about the needs of their patients for palliative care.

### Data analysis

Descriptive characteristics of the participants were presented using frequency and percentages for the survey tools. The semi-structured interview data was analyzed using a summative content analysis approach guided by *Hsieh and Shannon* [25]. The responses provided by the participants were systematically analyzed and grouped into general themes guided by the interview guide questions. These themes were subsequently categorized into sub-themes as represented in the result (Table 1). A comparison between children’s and adults’ needs for PC was done using the Chi-Squared test to address the third objective. Statistical analysis was performed using IBM SPSS Statistics for Windows, Version 21.0, and a *p*-value < 0.05 was considered statistically significant.

**Table 1:** Themes analysis: Assessment of the Patients’ Needs for Palliative Care

	Frequency	%
**Effects of palliative care on the patients**: *		
Helps to have a normal life Reliefs of pain and discomfort Die in peace	36 58 14	33.3 53.7 13
Specific needs are unmet in the population that you serve: *		
Special center Financial support Psychological/Emotional support Social support/Family care I don’t know	67 78 43 57 43	62 72.2 39.8 52.8 39.8
How can the needs be addressed? *		
Financial help Special centers Specialized care providers I don’t know	71 69 54 43	65.7 63.9 50 39.8
How dose the local policy supports the patients? *		
Medical support\provides pain killer Basic medical care Limited financial support I don’t know	59 32 13 27	54.6 29.6 12 25

*More than one response may have been recorded by each respondent.

## Results

Nurses made up approximately 66% of this sample, and the rest were physicians. Most of the physicians had 5–10 years of experience with a majority of the physicians being oncologic surgeons (Table 2).

**Table 2:** Demographic Characteristics of the Care Providers

	Frequency	%
**Profession**:		
Specialist Resident Nurse	31 5 72	28.7 4.6 66.7
**Specialty**:		
Oncology surgery Pediatric oncology Nurse specialist Bedside nurse	25 11 10 62	23.1 10.2 9.3 57.4
**Years of experience**:		
1–5 5 < – 10 10 <	21 69 18	19.4 63.9 16.7

The care providers’ experience in PC is represented in Table 3. None of the nurses were familiar with PC and were not able to describe it. They also indicated they had not participated in any activities related to this type of care. All the physicians reported experience with PC, however only 22% of them gave a correct and complete definition of PC with approximately 33% indicating that they participate in PC activities through patient care or training activities.

**Table 3:** Knowledge and Experience of Care Providers in Palliative Care

	Frequency	%
**Familiar with palliative care**:		
Yes No	36 72	33.3 66.7
**Given the meaning of palliative care by the providers (***n*** = 36)**:		
Correct and complete Incomplete Incorrect	8 23 5	22.2 63.9 13.9
**Participation in related activities (***n*** = 36)**:		
Yes No	12 24	33.3 66.7
**Receiving training in palliative care (***n*** = 36)**:		
Yes No	8 28	22.2 77.8

Four main themes (questions with Asterisk) and their subthemes are represented in Table 1. *(1) Effects of palliative care on the patients*; *(2) Specific needs are unmet in the population that you serve; (3) How can the needs be addressed?*; *(4) How does the local policy support the patients?* Table 4 shows providers’ responses regarding patients’ needs for PC. Most of the providers (either pediatric healthcare providers (PHCP) or adult healthcare providers (AHCP)) select neutral responses (53.8% and 48.4%) about the needs of patients for PC. While approximately 40% of the providers gave answers between strongly agree and agree about patient needs for PC (AHCP = 45.3%, PHCP = 38.5%) with no significant difference between the providers’ prospects in the two groups (*P* = 0.961).

**Table 4:** The patients need palliative care

The patients need palliative care	AHCP *n* = 95		PHCP *n* = 13		*P*
	Freq. %		Freq. %		
Strongly agree	13	13.7	2	15.4	
Agree	30	31.6	3	23.1	χ2 = 0.621 *P* = 0.961
Neutral	46	48.4	7	53.8	
Disagree	5	5.3	1	7.7	
Strongly disagree	1	1.1	0	0	

AHCP = Adult Health Care Providers, PHCP = Pediatric Heath Care Providers, χ2 = chi squair test with significance level ≤ 0.05.

When asked about adult or pediatric patient needs for palliative care, the most frequent needs include help with daily living activities (*n* = 95 and *n* = 13 respectively) and physical care during end-stage (*n* = 95 and *n* = 13 respectively). Regarding family/caregiver needs, the AHCP reports that the caregivers\families experience stress associated with financial and additional assistance needs (*n* = 95), while the PHCP reports the caregivers\families have psychological and physical needs (*n* = 13 and *n* = 13 respectively). (Table 5).

**Table 5:** The Need Assessment Tool (NAT)

Patient well-being		AHCP *n* = 95	PHCP *n* = 13
		*N* (%)*	*N* (%)*
1	Did the patients have concerns about spiritual or existential issues?	90(94.7)	11(84.6)
2	Did the patients have financial or legal concerns that are causing distress or require assistance?	92(96.8)	13(100)
3	Did the patients need help with daily living activities?	95(100)	13(100)
4	Are there health beliefs, cultural or social factors involving the patient or family that are making care more complex?	93(97.9)	13(100)
5	Are the patient’s psychological symptoms interfering with well-being or relationships?	94(98.9)	13(100)
6	Do the patients experience unresolved physical symptoms?	92(96.8)	12(92.3)
7	The patients would have indication of palliative care?	95(100)	13(100)
8	Do you think the patients need more pain killers during end-stage?	94(98.9)	12(92.3)
9	Do you think the end-stage patients need more comfort procedures?	91(95.8)	13(100)
10	Do you think the patients need more psychological support during end-stage?	93(97.9)	13(100)
11	Do you think the patients need more physical care during end-stage?	95(100)	12(92.3)
12	Do you think the current care provided to end-stage patients in this hospital is enough?	92 (96.8)	13 (100)
**Ability of caregiver/family to care for the patient**			
1	Does the caregiver or family have financial or legal concerns that are causing distress or require assistance?	95(100)	12(92.3)
2	Is the family currently experiencing problems that are interfering with their functioning or interpersonal relationships, or is there a history of such problems?	92(96.8)	13(100)
3	Is the caregiver or family having difficulty coping?	94(98.9)	13(100)
4	Is the caregiver or family having difficulty providing physical care?	93(97.9)	13(100)
5	Is the caregiver or family distressed about the patient’s physical symptoms?	93(97.9)	13(100)
**Caregiver well-being**			
1	Is the caregiver or family experiencing physical, practical, spiritual, existential, or psychological problems that are interfering with their well-being or functioning?	95(100)	12(92.3)
2	Is the caregiver or family experiencing grief over the impending or recent death of the patient that is interfering with their well-being or functioning?	94(98.9)	13(100)
**Total mean#**		**0.98**	**0.97**

* Yes response. M = Mean, SD = Standard deviation. AHCP = Adult Health Care Providers, PHCP = Pediatric Health Care Providers. **#** No statistical differences between mean.

## Discussion

This study identified the current access and availability of PC services, while the available literature identified the current services and future needs of PC in other geographic locations, including those in Northern Ireland, Egypt, Spain, and worldwide [26–28]. While each of these locations has unique nuances, the data gathered for each supports the expansion of PC services for the benefit of patients, their families, and their caregivers. There is no current literature that assessed the knowledge, interest, and perspectives of PC among healthcare providers and leaders in Upper Egypt, which is a gap that this study helped fulfill.

Results indicate that a vast majority of the providers represented were not familiar with PC services, had not received any training regarding PC, and had not participated in PC activities. A different study located at another hospital in Egypt corroborates a need for continuing education about PC for health care providers [29, 30]. Because it is a new concept and in its infancy stage in Egypt, there are no specialized courses in training or specialty in PC, which affects the delivery and accessibility of PC services. In the United States, where palliative care is a more developed and widely accessible form of care, there remains a need to educate health care providers and the community on palliative care [31]. The United States Congress introduced the Palliative Care and Hospice Education and Training Act **(**PCHETA) bill in 2023 to expand the palliative care workforce, research, education, interventions and public awareness, in an effort to change the current trends in PC and PC education [32]. This underscores similar need for curriculum changes within the medical and nursing education system in Egypt. The curriculum should include holistic education of aspects of PC including the principles and skills required for effective care delivery [31]. The World Health Organization (WHO) verifies that worldwide, medical training and healthcare policy has minimal if any inclusion of PC. The WHO also considered providing PC an ethical obligation of healthcare workers and the right of all humans to receive PC. The policy suggestions of the WHO are consistent with the suggestions of this study to increase PC education and make supportive systemic changes to support PC [26].

### Limitation

One limitation of this study is that it was conducted at a single medical facility in Egypt; therefore, study findings may not directly mirror results of the whole. In future research, it would be beneficial to conduct a more holistic assessment to understand the knowledge, interest, and perspective of PC among healthcare providers and healthcare leaders of Egypt and the Middle Eastern Region. In addition to the needs of providers, it would be beneficial to understand the needs of administrators and policymakers to improve PC access on a systemic level.

## Conclusion and recommendatation

The findings of this study suggest that healthcare providers and nurses, need training and education that focuses on identifying and explaining PC concepts and how they can be practiced in the clinical fields. Results highlight a gap in both PC knowledge and services, highlighting a need to provide PC. Additionally, a majority of providers require education and training regarding PC concepts. Changes in curriculum and policies are required to support implementation of PC for all adult and pediatric population.

### Electronic Supplementary Material

Below is the link to the electronic supplementary material.

Supplementary Material 1

## Data Availability

Any datasets used can be accessed through request to the corresponding author.
