# Supplementary material for: Health care providers’ perspectives on the need for palliative care in Upper Egypt: a descriptive exploratory study including children and adult patients
Source: BMC Palliat Care. 2024 Jun 15;23:152. doi: 10.1186/s12904-024-01469-5 (PMC11179283; doi:10.1186/s12904-024-01469-5)
Supplement: Supplementary file 1 — Supplementary Material 1 [file 12904_2024_1469_MOESM1_ESM.docx]

**SURVEY QUESTIONNAIRE**

**Protocol #: ___________________ Date: ____________**

**Participant ID: _______________**

**Survey 1: Sociodemographic data and Providers Knowledge about Palliative Care**

1. Please select your profession

Consultant  Nurse

Specialist.  Administrative

Resident  Privilege physician

Other

1. Please Mention your specialty: -----------------------------------
2. Years of experience in your specialty: --------------------------
3. For how long have you been working in this hospital: ------------------------

**Providers Knowledge about Palliative Care Semi-Structured Interview**

1. Are you familiar with the concept of palliative care?

Yes  No

If yes, please write what palliative care means to you:

1. Do you have experience or have participated in any activities related to palliative care?

Yes  No

If yes, please elaborate it:

1. Did you receive any training specific to palliative care?

Yes  No

If yes, please mention it:

1. Please select the response you most align with: I think patients need palliative care?

Strongly agree

Agree

Neutral

Disagree

Strongly Disagree

Please give rational for your answer

1. How do you think palliative care will affect the patients?
2. What specific needs are unmet in the population you serve?
3. How can these needs be addressed?
4. What barriers do your patient frequently cite?
5. What is done/how to overcome these barriers?
6. How is the local policy support the patients?
7. Are there any specific care or policy for end of life patients?

Yes  No

If yes, please mention:

**Survey 2: The Need Assessment Tool (NAT) Items:**

| **The Need Assessment Tool (NAT) Items** | | | |
| --- | --- | --- | --- |
| **Patient well-being** | | **Yes** | **No** |
| 1 | Did the patients have concerns about spiritual or existential issues? |  |  |
| 2 | Did the patients have financial or legal concerns that are causing distress or require assistance? |  |  |
| 3 | Did the patients need help with daily living activities? |  |  |
| 4 | Are there health beliefs, cultural or social factors involving the patient or family that are making care more complex? |  |  |
| 5 | Are the patient’s psychological symptoms interfering with well-being or relationships? |  |  |
| 6 | Do the patients experiencing unresolved physical symptoms? |  |  |
| 7 | The patients would have indication of palliative care? |  |  |
| 8 | Do you think the patients need more pain killer during end stage? |  |  |
| 9 | Do you think the end stage patients need more comfort procedure? |  |  |
| 10 | Do you think the patients need more psychological support during end stage? |  |  |
| 11 | Do you think the patients need more physical care during end stage? |  |  |
| 12 | Do you think current care provided to end stage patients in this hospital is enough? |  |  |
| **Ability of caregiver/family to care for the patient** | | | |
| 13 | Does the caregiver or family have financial or legal concerns that are causing distress or require assistance? |  |  |
| 14 | Is the family currently experiencing problems that are interfering with their functioning or inter-personal relationships, or is there a history of such problems? |  |  |
| 15 | Is the caregiver or family having difficulty coping? |  |  |
| 16 | Is the caregiver or family having difficulty providing physical care? |  |  |
| 17 | Is the caregiver or family distressed about the patient’s physical symptoms? |  |  |
| **Caregiver well-being** | | | |
| 18 | Is the caregiver or family experiencing physical, practical, spiritual, existential or psychological problems that are interfering with their well-being or functioning? |  |  |
| 19 | Is the caregiver or family experiencing grief over the impending or recent death of the patient that is interfering with their well-being or functioning? |  |  |
